# Stress, Isolation, and Sleep Quality among Breast Cancer Survivors throughout the COVID-19 Pandemic: A Longitudinal Study in a Multi-Ethnic Cohort

**DOI:** 10.21203/rs.3.rs-3231825/v1

**Published:** 2023-08-10

**Authors:** Fangyuan Zhao, Jincong Q. Freeman, Nora Jaskowiak, Gini F. Fleming, Rita Nanda, Diane S. Lauderdale, Olufunmilayo I. Olopade, Dezheng Huo

**Affiliations:** University of Chicago; University of Chicago; University of Chicago; University of Chicago; University of Chicago; University of Chicago; University of Chicago; University of Chicago

**Keywords:** breast cancer, insomnia severity index, longitudinal survey, sleep quality, social isolation, stress

## Abstract

**Purpose:**

This study examined how stress, isolation, and sleep quality were impacted throughout the COVID-19 pandemic among breast cancer survivors (BCS).

**Methods:**

BCS enrolled in the Chicago Multiethnic Epidemiologic Breast Cancer Cohort were surveyed in 2020, 2021, and 2022. An 11-item isolation/stress score was repeatedly measured in each survey and its changes were examined through mixed-effects models. Sleep quality was assessed in 2022 by the Insomnia Severity Index (ISI).

**Results:**

In total, 1899 BCS responded (response rate: 62.8%), of whom 69% were White and 24% Black (median time since diagnosis: 5.1 years, IQR: 2.3-9.2). The isolation/stress score decreased significantly from 2020 to 2022 for White BCS, but only started declining for Black BCS in 2022. Consequently, although there were no significant racial difference in 2020, Black BCS had significantly higher isolation/stress scores in 2021 and 2022 (*P* < .01), while it became nonsignificant after adjusting for socioeconomic factors. BCS who were single, on Medicaid, without a high school degree, or with annual household income <$35,000 had significantly higher isolation/stress scores. Regarding sleep quality, 48% of BCS reported clinically-significant insomnia (ISI ≥ 8), and insomnia was strongly associated with higher isolation/stress scores (*P*-trend < .001).

**Conclusions:**

Our findings suggested that the isolation/stress level improved among BCS as the pandemic subsided, but this positive trend was not observed equally across racial/ethnic groups potentially due to lack of resources.

**Implications for Cancer Survivors:**

Additional resources, such as access to counseling services and sleep assistance programs, might support the post-pandemic recovery of undersevered BCS.

## Background

The COVID-19 pandemic has had far-reaching consequences for individuals across the globe, with those having underlying medical conditions, such as cancer, being particularly vulnerable. Cancer patients had significantly higher risks of COVID-19 infection, hospitalization, and death [1]. The indirect effects of the pandemic extend beyond the virus itself, influencing various aspects related to the quality of life (QoL) of cancer patients and survivors, such as stress level and sleep quality.

The pandemic has been shown to increase stress, anxiety, and depressive symptoms among the general population [2–4]. Cancer patients and survivors may have faced additional stressors, such as delayed treatment, heightened concerns about infection risks, and limited access to follow-up care [5, 6]. The elevated stress levels could adversely affect their QoL and, consequently, long-term survival [7, 8]. Due to lockdowns and gathering restrictions, many cancer survivors could also experience loss of social support from their family, friends, and patient support groups, further impeding their ability to cope with stress [6, 9]. While many studies have reported increased levels of stress and isolation among cancer patients since the pandemic [5, 6, 10], the majority of them were conducted during the early phase of the pandemic, and the changes in levels of stress and isolation over time, particularly after vaccines became available, remained unclear.

Sleep quality, another crucial aspect of patient QoL, may have also been affected by the pandemic. We hypothesized that elevated stress levels and increased social isolation may compromise sleep quality [11]. However, some studies also suggested that certain conditions arising from the pandemic, including reduced commutes and increased schedule flexibility leading to fewer work hour constraints, could potentially enhance sleep quality [12, 13]. Therefore, further investigation is required as both positive and negative effects have been proposed [12–15].

In addition, it remains unclear whether cancer patients from racial/ethnic minority groups were disproportionally affected by the pandemic, exacerbating the well-documented racial disparities in breast cancer mortality [16–18]. Early studies have found that racial/ethnic minorities, particularly Black and Hispanic populations, were more likely to contract COVID-19, require hospitalization, and die from the disease [19–21]. More recent data suggest that this disparity has narrowed over time [22]. The reasons for these disparities can be complex, encompassing a wide range of socioeconomic status (SES) and structural factors [22]. Meanwhile, research investigating the racial disparities in cancer patient QoL during the pandemic has been scant [23].

In light of these knowledge gaps, we initiated a survey between July and September 2020 among a diverse cohort of breast cancer patients and survivors [24]. In the survey, we observed a significant increase in isolation and stress post-pandemic compared to pre-pandemic levels, with no significant racial differences found in post-pandemic isolation/stress levels [24]. To examine the evolving impact of the pandemic, we conducted two additional waves of surveys in 2021 and 2022. This allowed us to repeatedly measure isolation and stress levels during different phases of the pandemic. Additionally, we measured sleep quality in 2022 to compare with pre-pandemic data in literature and assess how sleep quality might be impacted by the pandemic.

## Methods

### Survey Design

This longitudinal survey study was conducted within the Chicago Multiethnic Epidemiologic Breast Cancer Cohort (ChiMEC) [25]. Initiated in 1993, ChiMEC consisted of over 4700 breast cancer patients diagnosed and treated at the University of Chicago Medicine, with most of them coming from the Chicago metropolitan area. Between July and September of 2020, 2021 and 2022, three waves of surveys were sent out to eligible ChiMEC participants via RedCap and follow-up phone calls [24]. The first two waves of surveys were sent out to all ChiMEC patients who consented to follow-up questionnaires at recruitment, while the third wave was only sent to patients who responded to at least one of the previous two waves of surveys and/or responded to the baseline survey at recruitment (**Supplemental Fig 1**). The surveys were sent out to 3023 patients in total, with an overall response rate of 62.8%.

### Measures

The three surveys repeatedly measured isolation and stress. An isolation/stress score (ranging from 0-44, higher score indicating worse isolation/stress level) was calculated from 11 individual question items adopted from existing item banks [26, 27]. The items of the total isolation/stress score showed good internal consistency in all three waves of surveys (Cronbach α= 0.85, 0.87, 0.89, respectively). The total score can be further decomposed into the social isolation score (4 items, ranging from 0-16; Cronbach α= 0.70, 0.74, 0.75, respectively) and the stress score (7 items, ranging from 0-28; Cronbach α= 0.81, 0.79, 0.79, respectively) to better understand factors associated with social isolation and stress, respectively. Scores were calculated when responses were provided for the majority of the corresponding question items.

To evaluated sleep quality, we measured insomnia severity and sleep time in the wave 3 survey in 2022. To measure insomnia severity, we applied the 7-item Insomnia Severity Index (ISI) [28]. To estimate sleep time, we adopted two self-report measures from the Pittsburgh Sleep Quality Index (PSQI)[29]: self-reported average sleep time and self-reported wake-bed time differences (i.e. duration calculated from self-reported wake-time, time-to-fall-asleep, and bed-time). Both measurements have been used previously [30]; one study among Black respondents reported that self-reported average sleep time was more concordant with actigraphy-based results [31].

Patient-reported race and ethnicity were recorded following the Centers for Disease Control and Prevention Race and Ethnicity codes. Clinical characteristics (i.e., date of birth, date of diagnosis, molecular subtype, tumor stage, comorbidities) and insurance type were extracted from electronic medical records and hospital cancer registry. Individual-level SES factors including marital status and education level were collected through questionnaires and/or electronic medical records. The survey also contained questions on the number of people living in the same household (wave 1) and annual household income (wave 2). To collect community-level SES of patients, their residential addresses were geocoded to identify their census block groups and were then linked to the American Community Survey (2016-2020) [32] and the Area Deprivation Index (ADI, 2020) [33, 34].

### Statistical Analysis

Standard descriptive statistics were used to compare patient characteristics, using t-tests for normally-distributed continuous variables, Wilcoxon rank-sum tests for skewed continuous variables and ordinal variables, and χ^2^ tests for categorical variables. Mixed-effects models were used to examine the changes in the isolation/stress scores across different waves of surveys and assess whether racial disparities existed in each year by including an interaction term between survey year and racial/ethnic groups, adjusting for potential confounding demographic, clinical, and SES characteristics. Ordinal logistic regression was used to examine the factors associated with ISI and estimate the odds ratios (OR) with 95% confidence intervals (CI). To address missing data in annual household income and education level, we conducted multiple imputation employing both individual-level demographic and clinical characteristics as well as the neighborhood-level SES characteristics. We utilized the “mi” command in Stata, which iteratively imputes missing values while accounting for the longitudinal structure of the data [35]. We then performed sensitivity analysis in the imputed datasets. Statistical analyses were conducted using the STATA 17.0 software package (StataCorp, College Station, TX).

## Results

In total, the study received responses from 1899 breast cancer patients and survivors, with 68.5% of them responding to at least two waves of surveys. Of the responders, 1317 (69.4%) were non-Hispanic White (“White”), 450 (23.7%) were non-Hispanic Black (“Black”), 53 (2.8%) were Hispanic, 75 (4.0%) were Asian and 2 (0.1%) were Native Americans. As numbers in racial/ethnic groups other than Black and White were small, most of the further analyses on racial disparities included Black and White patients. The median time from diagnosis to first survey was 5.1 years (IQR: 2.3-9.2), with only 7.8% of the patients diagnosed within 1 year before their first survey response.

Black patients were more likely to be diagnosed at older ages, with triple-negative breast cancer, at later stages, and with higher comorbidity burden (Table 1). In terms of the SES factors, Black patients were much more likely to be on Medicaid (18.0% vs. 1.4%; *P* < .001), be single or never married (28.2% vs. 6.3%; *P* < .001), have less education, and have lower levels of annual household income. Black patients were also more likely to live in neighborhoods with lower SES in terms of income and education level, which was also captured by more disadvantaged ADI.

### Social Isolation and Stress

**Figure 1:**
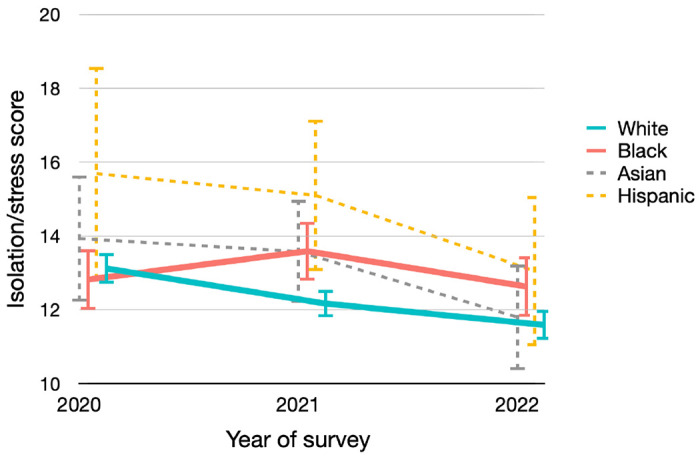
Total Isolation/Stress Score in Three Waves of Surveys by Different Racial/Ethnic Groups Change of isolation/stress score in the three waves of surveys stratified by different racial/ethnic groups (mean scores and 95% CIs were presented)

Overall, the isolation/stress level was moderate (Fig 1). For White patients, the average isolation/stress score significantly improved from 2020 to 2022, declining from 13.1 to 12.2 to 11.6 (*P*-trend < .001). As for Asian patients, despite a small sample size (n = 75), the isolation/stress score has also significantly improved (*P*-trend = .044). However, this positive trend was not seen for Black patients, with the average score in the three waves of surveys being 12.8, 13.6 and 12.6, respectively (*P*-trend = .84). Hispanic patients had the highest isolation/stress scores throughout the three waves of surveys, although the 95%CI was wide due to limited sample size (n = 53).

Delving deeper into the individual questions of the total isolation/stress score can help us pinpoint the exact areas where these disparities presented (**Supplemental Table 1**). White patients felt significantly more confident than Black patients in getting medical help and keeping up with work and home responsibilities in all three waves of surveys. On the other hand, Black patients were significantly more likely to feel isolated, overwhelmed, and worried about getting COVID in the 2021 survey, and were significantly more likely to worry about going to hospitals in 2021 and 2022. In addition, more patients on Medicaid expressed “Often” or “Always” being worried about the need to go to hospital compared to patients covered by private insurance in all three years (15.6% vs. 8.4% in 2020, 26.0% vs. 9.5% in 2021, and 18.2% vs. 7.1% in 2022; all *P* < .05).

Mixed-effects models showed similar findings as the descriptive analysis (Table 2). The isolation/stress score significantly decreased each year for White patients, while an improvement was only observed for Black patients in the last wave of survey and there was a statistically significant interaction between racial group and survey years (*P* < .001). The unadjusted differences in isolation/stress scores between Black and White patients was 1.34 (95%CI: 0.57, 2.10) in 2021 and 1.14 (95%CI: 0.34, 1.95) in 2022. Individual-level SES factors were significantly associated with both race/ethnicity and the isolation/stress score, and after adjustment of SES factors, the racial differences in the last two waves of surveys were not statistically significant. Patients on Medicaid had a higher isolation/stress score by 2.81 (95%CI: 1.39, 4.23) compared with those covered by private insurance. Patients who were younger, single or never married, without a high school degree, and with annual household income of <$35,000 also had significantly higher isolation/stress scores. No associations were found between the isolation/stress score and years since breast cancer diagnosis, tumor characteristics, or comorbidities.

Decomposing the total isolation/stress score into the social isolation score and the stress score found that they were associated with different individual-level SES factors (**Supplemental Table 2**). Specifically, the social isolation score was significantly associated with marital status and the number of people living in the same household, with patients being married and patients living with more people having significantly lower levels of social isolation. Whereas for the stress score, it was significantly associated with insurance type and education level. The racial differences observed in the two scores were no longer statistically significant after adjusting for the relevant individual-level SES factors.

### Sensitivity Analysis

To account for the missing values in annual household income and education level, we conducted multiple imputation. The main findings remained consistent in the imputed datasets (**Supplemental Table 3**). The isolation/stress score for White patients significantly decreased from 2020 to 2022 (*P* < .001), but no statistically significant changes were found for Black patients. The racial differences were not significant in all three waves of survey, after adjusting for the imputed annual household income in addition to age at diagnosis and years to first survey.

### Sleep Quality

Through examining the ISI, we found that more than 48% of the patients had clinically-significant insomnia (ISI ≥ 8). Of these, 16.2% of Black patients and 11.3% of White patients suffered from moderate to severe insomnia (ISI ≥ 15). As we hypothesized, more severe insomnia was significantly associated with higher isolation/stress scores in all three waves of surveys (**Supplemental Fig 2**). More severe insomnia was also strongly associated with younger age at survey (OR = 1.24 per 10-year decrease in age; 95%CI: 1.13, 1.36) and lower annual household income (OR = 4.50 [comparing <$35,000 to ≥$200,000]; 95%CI: 1.96, 10.17). In terms of responses to the individual ISI questions (**Supplemental Table 4**), Black patients aged <65 years were significantly more likely to report having difficulty in falling asleep (*P* = .022) and problems waking up too early (*P* = .029).

In terms of sleep time, the two measurement methods demonstrated moderate consistency (Pearson’s correlation = 0.57; concordance = 0.44), with self-reported average sleep time being around one hour shorter than self-reported wake-bed time differences (Table 3). Despite this discrepancy, our subsequent results were reported based on self-reported average sleep time, as both methods yielded similar conclusions. White patients had significantly longer hours of sleep than Black patients on average (7.0 vs. 6.3 hours/day, *P* < .001), with patients aged ≥65 years having slightly longer sleep time. Black patients were significantly more likely to sleep for less than 6 hours/day compared to White patients, after adjusting for age (OR = 3.24; 95%CI: 2.22, 4.72). Among patients younger than 65 years old, Black patients needed significantly longer time to fall asleep compared to White patients (30 vs. 20 minutes, *P* = .005), which was consistent with our observation from the analysis of individual ISI questions.

## Discussion

This longitudinal study among 1899 breast cancer patients and survivors from 2020 to 2022 found that their total isolation/stress levels improved during the pandemic, but not equally across racial/ethnic groups. In the first wave of survey in 2020, both Black and White patients had elevated isolation/stress levels compared to pre-pandemic times [24]. In the 2021 and 2022 surveys, the isolation/stress level for White patients monotonically decreased. However, Black patients did not exhibit this positive trend and experienced significantly higher levels of isolation/stress in 2021 and 2022 compared with White patients. Our findings suggest that, as the pandemic progressed and vaccines became available, Black and White patients experienced its effects differently and appeared to possess varying levels of resources to cope with these challenges.

The observed racial differences in isolation/stress scores can be attributed to the varying distributions of SES factors, like insurance type, education level, annual household income, and marital status across racial/ethnic groups. Compared to patients covered by private insurance, those with Medicaid had significantly higher isolation/stress scores and were more worried about the need to go to hospitals throughout the three waves of surveys. Patients who did not finish high school and those with annual household income of <$35,000 also had much higher isolation/stress scores, indicating their lack of resources to cope with the challenges posed by the pandemic [36, 37]. Recognition of how these SES factors can explain the racial disparities identified, and further understanding the underlying structural reasons is salient to addressing racial/ethnic disparities and promoting health equity [38]. Consistent with previous reports, no associations were observed between the isolation/stress level and tumor characteristics at diagnosis in our patient cohort [9, 39].

Further decomposing the total score into the social isolation score and the stress score, we found that patients who were married or living with more people had significantly lower social isolation level, consistent with previous studies emphasizing the importance of social support from family members [6, 9, 36]. According to prior qualitative studies, in addition to the reduced level of social support from family and friends since the pandemic, patients also reported a substantial loss of support received from healthcare providers and other cancer survivors [40, 41]. As we recover from the pandemic, we could envision increasing virtual and in-person social opportunities for patients and survivors to connect with each other and enhance their social support networks, providing an additional layer of support alongside that from family and friends.

Sleep quality was evaluated by measuring insomnia severity and sleep time. Adopting the widely used and validated ISI [28, 42], we found a mean ISI of 7.9 (SD = 5.5), which was similar with pre-pandemic findings among cancer survivors (mean ranging from 7-8; SD ranging from 5-7) [13, 42–44]. Using a cutoff of 8 [44], we found that over 48% of the patients were considered to have clinically-significant insomnia, which was also in line with pre-pandemic reports (ranging from 30% to 60%) [13, 42, 45]. Although the ISI levels did not appear elevated compared to pre-pandemic times, we did observe a significant correlation between elevated isolation/stress and more severe insomnia. In terms of sleep time, we found that Black patients were less likely to get sufficient sleep (i.e., ≥ 6 hours/day) and had more difficulty to fall asleep compared to their White counterparts. As the relationship between sleep duration and breast cancer survival remains unclear [46–49], we plan to conduct follow-up studies in our cohort to address this question.

The major strength of our study is its longitudinal design, which enables us to examine changes in the isolation/stress levels within a large group of patients during the pandemic. In contrast, most of the previous studies were cross-sectional and conducted at the beginning of the pandemic, limiting our understanding of the longer-term effects and differential experiences of breast cancer patients and survivors as the pandemic changed and progressed [6]. As all three waves of surveys were conducted from July to September, any seasonal effects were also controlled. Furthermore, through utilizing our detailed SES data and geocoding, we were able to better examine the underlying reasons of the racial disparities observed.

Despite these strengths, there were several limitations to be noted. First, as a single-institution study, our patient cohort was not nationally representative (e.g., had relatively higher levels of education and income). Secondly, as there were no existing instruments to measure COVID-associated isolation/stress, the measurement we used differs from those used in other studies. Nevertheless, the items we employed showed great internal consistency. Additionally, other studies, similar to our findings, reported moderate stress and anxiety levels [6, 10]. Another limitation was that our sleep quality questions were self-reported and only included in 2022, when the impact of the pandemic might have already lessened.

In conclusion, our longitudinal study of 1,899 breast cancer patients and survivors revealed that, despite demonstrating considerable resilience and experiencing improving isolation/stress levels overall as the pandemic subsides, patients from different racial/ethnic groups may have experienced the pandemic differently and had different coping capacities, potentially due to SES factors. The sleep quality of patients in 2022 was similar to pre-pandemic reports and follow-up studies will be conducted to further understand its long-term effects on QoL and survival outcomes.

## Figures and Tables

**Table 1.** Demographic, Clinical and Socioeconomic Characteristics of the Survey Respondents Stratified by Racial/Ethnic Groups

Factor, No. (%)	White Patients (n = 1317)	Black Patients (n = 450)	Others ^a^ (n = 132)	P ^b^
Age at Breast Cancer Diagnosis, mean (SD), years	54.3 (11.5)	55.8 (12.8)	48.2 (11.4)	.036
Years from Breast Cancer Diagnosis to First Survey, median (IQR), years	5.2 (2.2, 9.2)	5.4 (2.6, 9.3)	4.3 (2.1, 8.1)	.25
Breast Cancer Subtype				<.001
HR+/HER2−	688 (70.8)	188 (57.0)	62 (63.3)	
HR+/HER2+	103 (10.6)	31 (9.4)	18 (18.4)	
HR−/HER2+	45 (4.6)	30 (9.1)	3 (3.1)	
TNBC	136 (14.0)	81 (24.5)	15 (15.3)	
Missing ^c^	345	120	34	
AJCC Stage				.006
0	210 (16.9)	89 (20.8)	24 (19.7)	
I	597 (48.2)	163 (38.2)	50 (41.0)	
II	305 (24.6)	123 (28.8)	36 (29.5)	
III	117 (9.4)	45 (10.5)	11 (9.0)	
IV	10 (0.8)	7 (1.6)	1 (0.8)	
Missing	78	23	10	
Charlson Comorbidity Index				.001
0	1116 (88.7)	354 (81.9)	111 (88.8)	
I	62 (4.9)	30 (6.9)	7 (5.6)	
≥ 2	80 (6.4)	48 (11.1)	7 (5.6)	
Missing	59	18	7	
Insurance Type				<.001
Private Insurance	958 (72.7)	221 (49.1)	104 (78.8)	
Medicare	246 (18.7)	113 (25.1)	12 (9.1)	
Medicaid	19 (1.4)	81 (18.0)	6 (4.5)	
Others	94 (7.1)	35 (7.8)	10 (7.6)	
Education Level				<.001
Did not finish high school	6 (0.5)	11 (3.0)	1 (0.8)	
High school graduate/GED	107 (8.9)	43 (11.6)	12 (9.8)	
Trade/technical school, some college or Associate’s degree	249 (20.7)	130 (34.9)	15 (12.2)	
Bachelor’s degree	349 (29.0)	84 (22.6)	42 (34.1)	
Graduate or professional degree	493 (40.9)	104 (28.0)	53 (43.1)	
Missing	113	78	9	
Marital Status				<.001
Single/never married	82 (6.3)	124 (28.2)	17 (13.2)	
Married/living with a partner	966 (74.7)	172 (39.2)	93 (72.1)	
Divorced, separated, or widowed	245 (18.9)	143 (32.6)	19 (14.7)	
Missing	24	11	3	
No. of People Living With, median (IQR)				.002
Annual Household Income				<.001
< $15,000	13 (2.0)	27 (12.2)	5 (6.9)	
$15,000-$34,999	22 (3.3)	41 (18.5)	8 (11.1)	
$35,000-$49,999	40 (6.1)	28 (12.6)	6 (8.3)	
$50,000-$74,999	94 (14.3)	54 (24.3)	7 (9.7)	
$75,000-$99,999	83 (12.6)	34 (15.3)	11 (15.3)	
$100,000-$149,999	139 (21.1)	19 (8.6)	17 (23.6)	
$150,000-$199,999	112 (17.0)	13 (5.9)	6 (8.3)	
≥ $200,000	155 (23.6)	6 (2.7)	12 (16.7)	
Missing or prefer not to answer ^d^	659	228	60	
Neighborhood Annual Household Income, mean (SD)	103918 (46788)	62355 (33212)	94241 (44978)	<.001
% of Households with Annual Income Less Than $30,000 in Neighborhood				<.001
< 10	558 (43.9)	58 (13.7)	47 (37.0)	
10-20	391 (30.7)	85 (20.0)	40 (31.5)	
20-40	271 (21.3)	167 (39.4)	29 (22.8)	
≥ 40	52 (4.1)	114 (26.9)	11 (8.7)	
Missing	45	26	5	
% of Adult Population without a High School Degree in Neighborhood				<.001
< 1	296 (23.3)	40 (9.4)	22 (17.3)	
1-5	532 (41.8)	101 (23.8)	47 (37.0)	
5-10	260 (20.4)	105 (24.8)	24 (18.9)	
≥ 10	184 (14.5)	178 (42.0)	34 (26.8)	
Missing	45	26	5	
Area Deprivation Index (National Ranking Percentiles) ^e^				<.001
1^st^ quartile	528 (41.8)	28 (6.7)	43 (34.1)	
2^nd^ quartile	461 (36.5)	78 (18.5)	50 (39.7)	
3^rd^ quartile	213 (16.9)	185 (43.9)	23 (18.3)	
4^th^ quartile	61 (4.8)	130 (30.9)	10 (7.9)	
Missing	54	29	6	

Abbreviations: HR, hormone receptor; HER2, human epidermal growth factor receptor 2; TNBC, triple-negative breast cancer; AJCC, American Joint Committee on Cancer; GED, General Educational Diploma; IQR, interquartile range; SD, standard deviation.

a Other patients include 75 Asian patients, 53 Hispanic patients and 2 Native American patients.

b *P* values for the comparison between White patients and Black patients (excluding missing values) were estimated using t-tests for age at diagnosis; χ2 tests for tumor subtype, tumor stage, CCI, insurance type and marital status; Wilcoxon Rank-Sum tests for years from diagnosis to first survey, education level, and annual household income. To adjust for clustering of neighborhoods, the *P* values for the comparison between White patients and Black patients were estimated using linear regression for neighborhood annual household income and ordinal logistic regression for the other neighborhood level factors, all adjusting for clusters in census block group.

c HER2 was not tested in stage 0 patients so there are large numbers of missing data in subtypes.

d Household income was only asked in the wave 2 survey and some preferred not to answer, so there are large numbers of missing data.

e The percentiles were constructed by ranking the ADI from low to high for the nation and grouping the block groups/neighborhoods into bins corresponding to each 1% range of the ADI, with those in the first percentile being the least disadvantaged, and those in the hundredth being the most disadvantaged [33, 34].

**Table 2.** Mixed-effects Models of Total Isolation/Stress Score with Interactions between Survey Year and Race/Ethnicity

	Model 1 ^a^	Model 2	Model 3
	Coefficient (95% CI)	Coefficient (95% CI)	Coefficient (95% CI)
**Difference between Black vs. White patients by survey year**			
2020	−0.29 (−1.06, 0.48)	−0.94 (−1.87, −0.013) *	−1.25 (−2.46, −0.036) *
2021	1.34 (0.57, 2.10) **	0.39 (−0.49, 1.27)	0.24 (−0.84, 1.31)
2022	1.14 (0.34, 1.95) **	0.28 (−0.64, 1.19)	0.23 (−0.95, 1.41)
**Change of total score in White patients**			
2021 vs. 2020	−1.10 (−1.45, −0.74) ***	−1.11 (−1.48, −0.74) ***	−1.03 (−1.48, −0.58) ***
2022 vs. 2021	−0.62 (−0.97, −0.27) **	−0.59 (−0.95, −0.23) **	−0.77 (−1.21, −0.33) **
**Change of total score in Black patients**			
2021 vs. 2020	0.53 (−0.13, 1.20)	0.22 (−0.50, 0.93)	0.45 (−0.40, 1.30)
2022 vs. 2021	−0.81 (−1.48, −0.15) *	−0.70 (−1.39, −0.011) *	−0.77 (−1.58, 0.035)
**Age at breast cancer diagnosis, per 5-year increase**		−0.45 (−0.61, −0.29) ***	−0.34 (−0.51, −0.17) ***
**Years from breast cancer diagnosis to survey, per 1-year increase**		−0.022 (−0.082, 0.038)	−0.035 (−0.11, 0.038)
**Marital status**			
Married/living with a partner		1 (ref.)	
Single/never married		1.56 (0.55, 2.57) **	
Divorced, separated, or widowed		0.61 (−0.14, 1.36)	
**Insurance type**			
Private Insurance		1 (ref.)	
Medicare		1.06 (0.10, 2.02) *	
Medicaid		2.81 (1.39, 4.23) ***	
Others		0.42 (−1.50, 2.33)	
**Education level**			
Graduate or professional degree		1 (ref.)	
Bachelor’s degree		0.16 (−0.55, 0.87)	
Trade/technical school, some college, Associate’s		−0.30 (−1.06, 0.47)	
High school graduate/GED		−0.77 (−1.79, 0.26)	
Did not finish high school		5.00 (2.07, 7.93) **	
**Annual household income level**			
≥ $200,000			1 (ref.)
$100,000-$199,999			0.002 (−1.10, 1.10)
$35,000-$99,999			0.093 (−1.04, 1.22)
< $35,000			3.31 (1.80, 4.82) ***

Abbreviations: CI, confidence interval; GED, General Educational Diploma; ref.; reference.

* *P* < .05,

** *P* < .01,

*** *P* < .001

a Model 1, 2 and 3 were random-efffects models with the response variable being total isolation/stress score, and the predictors being the variables with their coeffieint estimates reported in the table, including an interaction between survey year and racial/ethnic groups.

**Table 3.** Responses to Sleep Quality Related Questions among Black and White patients Stratified by Age at Survey

Factor, No. (%)	Age at survey < 65 years old ^a^			Age at survey ≥ 65 years old		
	White Patients (n = 489)	Black Patients (n = 118)	*P* ^b^	White Patients (n = 390)	Black Patients (n = 123)	*P* ^b^
ISI, mean (SD)	8.2 (5.5)	8.9 (6.0)	.23	7.3 (5.1)	8.0 (6.0)	.24
ISI ^c^			.34			.30
No clinically significant insomnia (0-7)	238 (49.9)	54 (47.0)		209 (56.5)	60 (52.6)	
Subthreshold clinical insomnia (8-14)	177 (37.1)	40 (34.8)		128 (34.6)	38 (33.3)	
Clinical insomnia (moderate to severe) (15-28)	62 (13.0)	21 (18.2)		33 (8.9)	16 (14.1)	
Self-reported average sleep time, hours/day ^d^			<.001			.001
< 6	51 (10.4)	30 (25.6)		31 (8.0)	28 (23.1)	
6-7	140 (28.5)	46 (39.3)		82 (21.1)	30 (24.8)	
7-8	166 (33.8)	27 (23.1)		138 (35.5)	32 (26.4)	
8-9	107 (21.8)	10 (8.5)		101 (26.0)	26 (21.5)	
> 9	27 (5.5)	4 (3.4)		37 (9.5)	5 (4.1)	
Self-reported average sleep time, hours/day, mean (SD)	6.9 (1.2)	6.1 (1.3)	<.001	7.1 (1.2)	6.5 (1.4)	<.001
Self-reported wake-bed time differences, hours/day ^e^			<.001			.13
< 6	28 (5.8)	28 (26.9)		15 (4.0)	13 (12.0)	
6-7	65 (13.5)	21 (20.2)		36 (9.7)	19 (17.6)	
7-8	177 (36.8)	32 (30.8)		120 (32.3)	24 (22.2)	
8-9	153 (31.8)	12 (11.5)		133 (35.8)	27 (25.0)	
> 9	58 (12.1)	11 (10.6)		68 (18.3)	25 (23.1)	
Self-reported wake-bed time differences, hours/day, mean (SD)	7.8 (1.2)	6.9 (1.7)	<.001	8.1 (1.2)	7.9 (1.7)	.29
Time to fall asleep, minutes, median (IQR)	20.0 (10.0, 30.0)	30.0 (15.0, 45.0)	.005	15.0 (10.0, 30.0)	20.0 (10.0, 30.0)	.37

Abbreviation: ISI, Insomnia Severity Index; SD, standard deviation; IQR, interquartile range.

a The results were presented stratifying by age at survey to address the significant association between age and sleep time

b *P* values for the comparison between White patients and Black patients were calculated using t-tests for continuous sleep time and ISI, and Wilcoxon Rank-Sum tests for the other ordinal variables

c ISI categories were based on the guidelines presented previously [28]

d Self-reported average sleep time was based on responses to the question “During the past month, how many hours of actual sleep did you get at night? (this might be different than the number of hours you spent in bed.)”

e Self-reported wake-bed time differences was calculated based on responses to the questions “During the past month, when have you usually gone to bed at night?”, “During the past month, how long (in minutes) has it usually take you to fall asleep each night?” and “During the past month, when have you usually gotten up in the morning?”

## Data Availability

The de-identified data of the current study could be made available from the corresponding author (Dr. Dezheng Huo: dhuo@bsd.uchicago.edu) upon reasonable request and in compliance with the guidelines set by the University of Chicago Institutional Review Board.
